# P-1571. Risk Factors for Infections after Urological Procedures among Patients with Negative Urine Culture Screening

**DOI:** 10.1093/ofid/ofae631.1738

**Published:** 2025-01-29

**Authors:** Nutnicha Tantiwattanapaibul, Anucha Apisarnthanarak, Patranuch Noppakulsatit, Chatchawet Liwrotsap, Teerayut Tangpaitoon, Valeerat Swatesutipun, Dollacha Vanichkarn, Natthapitch Tangkaew, Kittiya Jantarathaneewat, Nuntra Suwantarat

**Affiliations:** Faculty of Medicine, Thammasat University, Pathum Thani, Thailand., Maung, Pathum Thani, Thailand; Thammasat University Hospital, Pratumthani, Pathum Thani, Thailand; Faculty of Medicine, Thammasat University, Pathum Thani, Thailand., Maung, Pathum Thani, Thailand; Faculty of Medicine, Thammasat University, Maung, Pathum Thani, Thailand; Faculty of Medicine, Thammasat University, Maung, Pathum Thani, Thailand; Faculty of Medicine, Thammasat University, Maung, Pathum Thani, Thailand; Factulty of Medicine, Thammasat Univeristy, Maung, Pathum Thani, Thailand; Faculty of Medicine, Thammasat University, Maung, Pathum Thani, Thailand; Faculty of Pharmacy, Thammasat University, Pathum Thani, Thailand., Maung, Pathum Thani, Thailand; Chulabhorn International College of Medicine, Thammsat University Hospital, Thammasat University, Maung, Pathum Thani, Thailand

## Abstract

**Background:**

The increased demand for urological procedures had become significant complications and correlating with an elevated prevalence of multidrug-resistant organisms (MDROs) infections, prolonged hospital stay, and the cost of treatment.

Table 1

**Figure:**
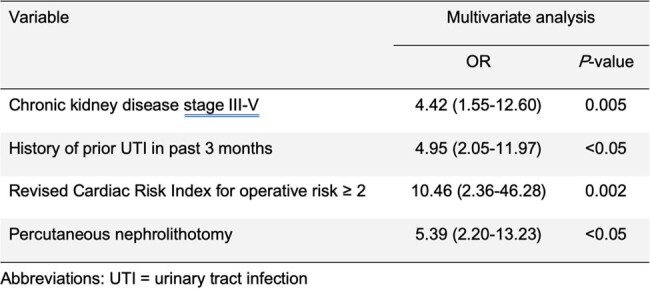

Multivariate analysis of risk factors for infection after urological procedures among patients with negative urine culture screening

**Methods:**

We conducted a retrospective cohort study to identify the risks for infections after urological procedures among patients with negative urine culture screening. The patients underwent transrectal ultrasound (TRUS) and biopsy of the prostate, transurethral resection of the prostate (TURP), holmium laser enucleation of the prostate (HoLEP), percutaneous nephrolithotomy (PCNL), ureteroscopy (URS), retrograde intrarenal surgery (RIRS), and transurethral resection of bladder tumor (TURBT) between 2021 and 2023, at Thammasat University Hospital, Thailand.

**Results:**

A total of 441 patients had the procedures. There were 55 patients (12.5%) developed infections including urinary tract infections (UTIs) (55/55; 100%) and sepsis (9/55; 16%). The significant risks of infections were chronic kidney disease (CKD) stage III-V (*P*=0.005), history of previous UTI within 3 months (*P*< 0.05), Revised Cardiac Risk Index (RCRI) for preoperative risk > 2 (*P*=0.002), and PCNL (*P*< 0.05). The patients received antibiotic prophylaxis with levofloxacin (26.5%) for TRUS and biopsy of the prostate and cefoxitin (73.5%) for other procedures. The most common causative pathogens of UTIs after the procedures were *Escherichia coli* (27/55; 49%), *Pseudomonas aeruginosa* (9/55; 16.4%) and *Klebsiella pneumoniae* (7/55; 12.7%). MDROs were identified including extended-spectrum cephalosporinase (24/55; 43.6%) and fluoroquinolone-resistant Gram-negative bacteria (12/55; 21.8%). The median hospital stay was 12 days (IQR 10-15) VS 3 days (IQR 3-4) for infection VS non-infection.

**Conclusion:**

Our study emphasized the novel significance of CKD and RCRI scores as risk factors and severity indices for preoperative risk assessment, correlating with postoperative infections following urological proceOur study emphasized the novel significance of CKD and RCRI scores as risk factors and severity indices for preoperative risk assessment, correlating with postoperative infections following urological procedures.

**Disclosures:**

**All Authors**: No reported disclosures

